# Salivary proteome and glucose levels are related with sweet taste sensitivity in young adults

**DOI:** 10.1080/16546628.2017.1389208

**Published:** 2017-10-30

**Authors:** L. Rodrigues, G. Costa, C. Cordeiro, C. Pinheiro, F. Amado, E. Lamy

**Affiliations:** ^a^ Instituto de Ciências Agrárias e Ambientais Mediterrânicas (ICAAM), Universidade de Évora, Évora, Portugal; ^b^ Centro de Química e Bioquímica, Faculdade de Ciências, Universidade de Lisboa, Lisboa, Portugal; ^c^ Departamento de Zootecnia, Escola de Ciências e Tecnologia, Universidade de Évora, Évora, Portugal; ^d^ Departamento de Química, Universidade de Aveiro, Aveiro, Portugal; ^e^ Química Orgânica, Produtos Naturais e Agro-Alimentares (QOPNA), Universidade de Aveiro, Aveiro, Portugal

**Keywords:** Sweet taste sensitivity, salivary proteome, salivary glucose, body mass index, amylase, carbonic anhydrase VI, cystatins

## Abstract

Sweet taste plays a critical role in determining food preferences and choices. Similar to what happens for other oral sensations, individuals differ in their sensitivity for sweet taste and these inter-individual differences may be responsible for variations in food acceptance. Despite evidence that saliva plays a role in taste perception, this fluid has been mainly studied in the context of bitterness or astringency. We investigated the possible relationship between sweet taste sensitivity and salivary composition in subjects with different sucrose detection thresholds. Saliva collected from 159 young adults was evaluated for pH, total protein concentration and glucose. One- and bi-dimensional electrophoresis (2-DE) were performed and protein profiles compared between sweet sensitivity groups, with proteins that were differently expressed being identified by MALDI-FTICR-MS. Moreover, Western blotting was performed for salivary carbonic anhydrase VI (CA-VI) and cystatins and salivary amylase enzymatic activity was assessed in order to compare groups. Females with low sensitivity to sweet taste had higher salivary concentrations of glucose compared to those with sensitivity. For protein profiles, some differences were sex-dependent, with higher levels of α-amylase and CA-VI in low-sensitivity individuals and higher levels of cystatins in sensitive ones for both sexes. Body mass index was not observed to affect the association between salivary proteome and taste sensitivity. To our knowledge, these are the first data showing an association between sweet taste and saliva proteome.

## Introduction

Sweet taste is associated with innate acceptance and preference for foods, functioning as a way of signalling energetic nutrients, namely carbohydrates [1]. The preference for sweet and high-fat foods is frequently reported as being associated with obesity development: obese people appear to have higher preferences for sweet and fat foods and their positive response to fat becomes even greater when sweetness is added to fatty foods [2]; moreover, different authors refer to the association between sweet taste perception and body weight [3,4], with sweet detection/recognition thresholds being reported as significantly lower in morbidly obese adolescents [5].

There is growing evidence that saliva composition is related to the way in which food is perceived in the oral cavity, namely with individuals’ taste responsiveness [6,7]. However, most of the studies on the influence of saliva protein composition on oral perception have been made for astringency perception [e.g. [8]] or for bitter taste [e.g. [9, 10]]. Few studies have attempted to relate the levels of salivary constituents to the perception of sweet taste. A recent study suggested the existence of a relationship between salivary amylase concentration and sweet taste scores in children [11]. However, to our knowledge, there are no studies linking the salivary proteome with sensitivity to sweet taste. One possible explanation for this is the assumption, by different authors, that results obtained with the bitter compounds phenylthiocarbamide (PTC) and 6-n-propylthiouracyl (PROP) could be extrapolated to other tastes, since responsiveness to these compounds has been widely accepted as a ‘marker’ of sensitivity for tastes and oral sensations in general [12]. However, such a relationship is questionable [13] and only a weak link between PROP taste sensitivity and sweet perception has been observed [14].

The objective of the present study is to evaluate the relationship between sensitivity to sweet taste and salivary composition, particularly protein profile and glucose levels, in normal weight and overweight young adults.

## Material and methods

### Subjects

Non-trained volunteer students from the University of Évora (*n* = 159), aged 18–30 years, were randomly assigned to the study. Some days before the tests volunteers answered different questions about their general and oral health, including allergies and chronic diseases. Only those that were healthy (no allergies and without chronic diseases), apparently free from oral or nasal disease and not taking medications that interfered with taste or odour perception were allowed to participate. All subjects were asked to take breakfast and refrain from eating and drinking anything, except water, for at least 1 h before testing. All tests were carried out in the morning, between 10.30 am and 12.00 pm. In order to classify their taste perception, each subject was tested twice in different visits, separated by a minimum 15 d and a maximum of 21 d. Potential changes in each subject’s oral and general health status were checked for their inclusion in the study.

Body mass index (BMI) was assessed by weighing and measuring each individual. World Health Organization (WHO) criteria were used for classifying individuals and defining them as normal weight (18 < BMI < 25 kg/m^2^) or overweight (BMI > 25 kg/m^2^).

All subjects read and signed an informed consent form. All procedures were in accordance with the Declaration of Helsinki for Medical Research involving Human Subjects and were approved by the institutional ethics committee from the University of Évora.

### Taste stimuli, sensory procedure and saliva collection

The taste stimuli were prepared on the day before the tests by dissolving sucrose in distilled water, storing it in a refrigerator and bringing it to room temperature before tasting.

At the beginning of the session, subjects received distilled water to rinse their mouths. Subsequently, they were instructed to not swallow for 5 min, and to collect all of the saliva formed in their mouths during that time by direct passive draining into an ice-cold collection tube maintained on ice during collection and until laboratory arrival. After that tubes were kept at −20°C.

For sweet taste threshold determination, serial concentrations of eight sucrose solutions were tested, in ascending order (0.34; 0.55; 0.94; 1.56; 2.59; 4.32, 7.20 and 12.0 g/L, according to standard ISO 3972). Individuals were asked to taste each sucrose solution, to compare it with distilled water and to note whether they were similar or different. The lowest solution identified as different from water was considered the detection threshold. These thresholds were taken into consideration to classify individuals in two groups: sensitive to sweet, when individuals had thresholds lower than the median of the thresholds observed; low-sensitive when thresholds were equal to or higher than the median.

### Salivary glucose levels, total protein concentration and pH

Salivary glucose levels were estimated using the colorimetric kit Glucose (GO) Assay (Sigma-Aldrich, Inc.), which is based on glucose oxidase reaction. The original protocol supplied by the manufacturer was adapted for microplates, with the reagents added in a volume proportional to the one recommended for each of them, reaching a final volume of 280 uL per well. Standards with the concentrations 2.5–30 µg/mL glucose were run in each microplate. Samples and standards were run in triplicate. Absorbance values were measured at 560 nm in a microplate reader (Glomax, Promega).

Total protein concentration was determined using the Bradford method. The pH of saliva samples was measured using a calibrated pH meter (HI 110 series, Hanna Instruments) and recording to two decimal places.

### Salivary protein composition

Salivary protein composition was studied, in general, by uni- and bi-dimensional electrophoretic profile and particularly for the proteins α-amylase, carbonic anhydrase VI and cystatins, by studying enzymatic activity and expression level by Western blot, respectively.

#### SDS PAGE electrophoresis

Samples from 33 females (20 sensitive and 13 low-sensitive) and 41 males (19 sensitive and 22 low-sensitive) were subjected to SDS PAGE. Each sample was run in triplicate. A total of 7.5 ug protein from each individual saliva sample was run on each lane, in a 12% polyacrylamide mini-gel (Protean xi, Bio-Rad), using a Laemmli buffer system [15]. An electrophoretic run was performed at a constant voltage of 150 V until the front dye reached the end of the gel. Gels were fixed for 1 h in 40% methanol/10% acetic acid, followed by staining for 2 h with Coomassie Brilliant Blue (CBB) G-250. Gel images were acquired using a scanning Molecular Dynamics densitometer with internal calibration and LabScan software (GE Healthcare), and images were analysed using Gel Analyzer software (GelAnalyzer 2010a by Istvan Lazar, www.gelanalyzer.com). Molecular masses were determined in accordance with molecular mass standards (Bio-Rad Precision Plus Protein^TM^ Dual Color 161-0394) run with protein samples.

#### Two-dimensional electrophoresis (2-DE)

For the two-dimensional protein profiles, 50 samples were analysed in duplicate, taking into account each sex individually and each experimental group of sweet taste. Gels from 23 samples of females (11 sensitive and 12 low-sensitive) and 27 of males (14 sensitive and 13 low-sensitive) were considered for analysis.

For 2-DE [16], saliva samples, in a volume corresponding to 175 µg total protein, were desalted and concentrated using 3 kDa cut-off ultra-filtration microfuge tubes (Nanosep 3K omega, PALL Corporation). After that proteins were subjected to isoelectric focusing in 13 cm pH 3–10NL IPG strips, for a total of 30 kVh, followed by vertical separation in 12% polyacrylamide gels, following the protocol described elsewhere [17]. Gels were fixed with 20% methanol, 10% acetic acid, stained with 0.1% CBB-G250, and destained with several washes of distilled water. Digital images of the 2-DE gels were acquired using the same procedure as that described for SDS-PAGE image acquisition. Gel analysis was performed using Image Master Platinum v.7 software (Amersham Biosciences, Europe GmbH, Freiburg, Germany), with automatic spot detection, followed by manual editing for spot splitting and noise removal.

### Protein identification by MALDI FTICR MS

#### Tryptic digestion

Bands or spots of interest were manually excised from gels, washed three times with 50% acetonitrile (ACN) (15 min for each wash) and once with 100% ACN (15 min). Dried gel pieces were incubated for 45 min with 10 mM DTT at 56°C, followed by incubation with iodoacetamide for 30 min in the dark and at room temperature. After washing with 50% ACN (15 min) and ACN 100% (30 min), gel pieces were dried in a SpeedVac. Fifteen µL of 6.7 ng/µL of porcine trypsin (Sequencing Grade Modified Trypsin, Promega) 50 mM ammonium hydrogencarbonate were added and incubation performed for 45 min at 4°C. Excess liquid was removed and 50 µL of 50 mM ammonium hydrogen carbonate was added to gel pieces and incubation was performed overnight at 37°C. Extraction of tryptic peptides was performed by addition of 10% of formic acid (FA)/50% ACN three times lyophilised in a Savant™ SpeedVac (Thermo Fisher).

#### Mass spectrometry

To identify target proteins, peptide mixtures were analysed by MALDI-FTICR-MS in a Bruker Apex Ultra, Apollo II combi-source (Bruker Daltonics, Bremen, Germany), with a 7 Tesla magnet (Magnex Corporation, Oxford, UK) as previously described [18,19]. Briefly, samples were desalted and concentrated using reverse phase Poros R2 (Applied Biosystems) and eluted directly to the MALDI target AnchorChip (BrukerDaltonics, Bremen, Germany) with the appropriated matrix, according to the manufacturer’s instructions. Matrix solution of α-cyano-4-hydroxycinnamic acid (CHCA; Fluka) was prepared at a concentration of 10 μg/μL in 50% ACN with 0.1% TFA. Monoisotopic peptide masses were determined using the SNAP 2 algorithm in Data Analysis software version 3.4 (BrukerDaltonics). External calibration was performed using the BSA tryptic digest spectrum, processed and analysed with Biotools 3.1 (BrukerDaltonics, Bremen, Germany).

#### Database search

Monoisotopic peptide masses were used to search for protein identification with Mascot software (Matrix Science, UK). The Swiss-Prot non-redundant protein sequence database (accessed in June 2014) was used for all searches. A minimum mass accuracy of 10 ppm, one missed cleavage in peptide masses, carbamidomethylation of Cys and oxidation of Met, as fixed and variable amino acid modifications, respectively, were considered. Criteria used to accept the identification were significant homology scores achieved in Mascot, significant sequence coverage values, and similarity between the protein molecular mass calculated from the gel and for the identified protein.

### Salivary α-amylase enzymatic activity

Salivary α-amylase enzymatic activity was performed according to Salimetrics® α-Amylase Kinetic Enzyme Assay Kit in a total of 87 samples [46 males (22 sensitive and 24 low-sensitive) and 41 females (20 sensitive and 21 low-sensitive)]. This method utilizes a chromogenic substrate, 2-chloro-p-nitrophenol linked with maltotriose. Salivary α-amylase, in this enzymatic action, reacted with substrate and yields 2-chloro-p-nitrophenol (PNP), which can be spectrophotometrically measured at 405 nm. The protocol was performed in 96-well plates, in accordance with the manufacturer’s instructions. Enzyme activity was expressed as the number of moles of PNP formed per minute, per l of saliva (U/L).

### Western blotting

Western blotting was used for comparison of expression levels of CA-VI and cystatins. Samples from the individuals run in SDS PAGE profiles were analysed in triplicate. After protein separation by SDS PAGE (5 μg total protein from each sample), in 14% polyacrylamide gels (100 V constant voltage) (mini-protean apparatus, Bio-Rad), proteins were transferred to a PDVF membrane by electroblotting using a Tris-glycine buffer system. After transferring, blocking was performed with 5% non-fat dry milk in TBS-Tween 20, for 2 h, with agitation, at room temperature. The membrane was cut, with the upper part incubated with primary antibody anti-CA-VI (Santa Cruz Biotechnology sc-99173; dilution: 1:200) and the lower part with primary antibody anti-cystatin S-SA-SN (Santa Cruz Biotechnology sc-73884; dilution: 1:200), overnight at 4°C. CA-VI and cystatin bands were detected with an alkaline phosphatase-linked secondary antibody (anti-rabbit and anti-mouse, respectively, GE Healthcare, 1:10,000 dilution), using a chemifluorescent substrate (ECF Plus Western Blotting Detection Reagents, GE, Healthcare). Membranes were revealed in a transilluminator (Gel-Doc, Bio-Rad) and a semi-quantitative analysis of band expression was carried out using the software Bio-Rad Image Lab 5.2.1.

### Statistical analysis

All data were analysed using descriptive statistics, and normality and homoscedasticity were evaluated using the Kolmogorov-Smirnoff and Levene tests, respectively. To assess the existence of differences between sexes and BMI, in terms of the proportion of individuals belonging to each taste perception group, a Chi-square test was performed. For the comparison of salivary parameters (individual levels of expression of protein bands, glucose concentration and α-amylase salivary enzymatic activity), a Student’s *t*-test or non-parametric equivalent (Mann-Whitney) was performed. Spearman’s correlation coefficient was used to verify the relationship between sucrose thresholds and salivary parameters. For the two-dimensional profiles, the volume percentages of the protein spots were tested using ANOVA-GLM with two fixed factors (taste sensitivity group and sex or taste sensitivity group and BMI). Bonferroni correction was used for multiple comparison. Statistical significance was considered as *p* < 0.05. All statistical analysis procedures were achieved using the SPSS 21.0 software package (SPSS Inc., Chicago, USA).

## Results

### Taste sensitivity according to sex and BMI

No differences were observed between normal weight and overweight in the percentage of individuals belonging to each sweet taste sensitivity group (Table 1).

**Table 1.** Percentage of normal weight and overweight individuals belonging to each sweet taste sensitivity group.

		% individuals	
	IMC (kg/m^2^)	«25	>25
Sweet taste sensitivity group	Sensitive	49.1	51.5
	Low-sensitive	50.9	48.5

Comparing males and females a tendency (*p* = 0.051) for higher mean sucrose detection thresholds, i.e. lower sensitivity, was observed in males (6.78 ± 0.45 g/L) than in females (5.60 ± 0.34 g/L). The Chi-square test showed no differences between sexes in the proportion of individuals belonging to each sweet taste sensitivity group. However, considering only the normal weight individuals, a tendency was observed for a higher percentage of females being sensitive to sweet, compared to males (*p* = 0.160): females, 54.1% sensitive and 45.9% low-sensitive; males, 40.5% sensitive and 59.5% low-sensitive.

### Salivary glucose levels

Although no significant differences were observed, if we consider both sexes together when analysing each sex separately, it was possible to note that females who are low-sensitive to sweetness have higher salivary glucose values (6.01 ± 1.43 µg/mL) than the sensitive ones (2.57 ± 0.33 μg/mL) (*p* = 0.015). Moreover, in females, glucose concentration presented a moderately positive correlation with sucrose thresholds (*n* = 39, R = 0.383, *p* = 0.016).

In terms of BMI, salivary glucose levels did not differ between normal weight (3.26 ± 0.27 μg/mL) and overweight (3.14 ± 0.52 μg/mL) (*p* = 0.826) individuals.

### Saliva composition

#### Saliva flow rate, pH and total protein concentration

Individuals with different sweet taste sensitivities did not diverge in their salivary flow rate, salivary total protein concentration and salivary pH (Table 2).

**Table 2.** Comparison of salivary parameters (mean ± standard error) between sweet taste sensitivity groups.

Salivary parameters (mean ± standard error)^a^			
	Sensitive	Low-sensitive	P
Total protein concentration (µg/mL)	446.07 ± 21.55	458.98 ± 19.95	0.662
Saliva flow rate (mL/min)	0.52 ± 0.03	0.57 ± 0.03	0.191
pH	7.60 ± 0.08	7.64 ± 0.11	0.774

^a^These values refer to the total of individuals under study.

#### Protein profile

**Figure 1.**
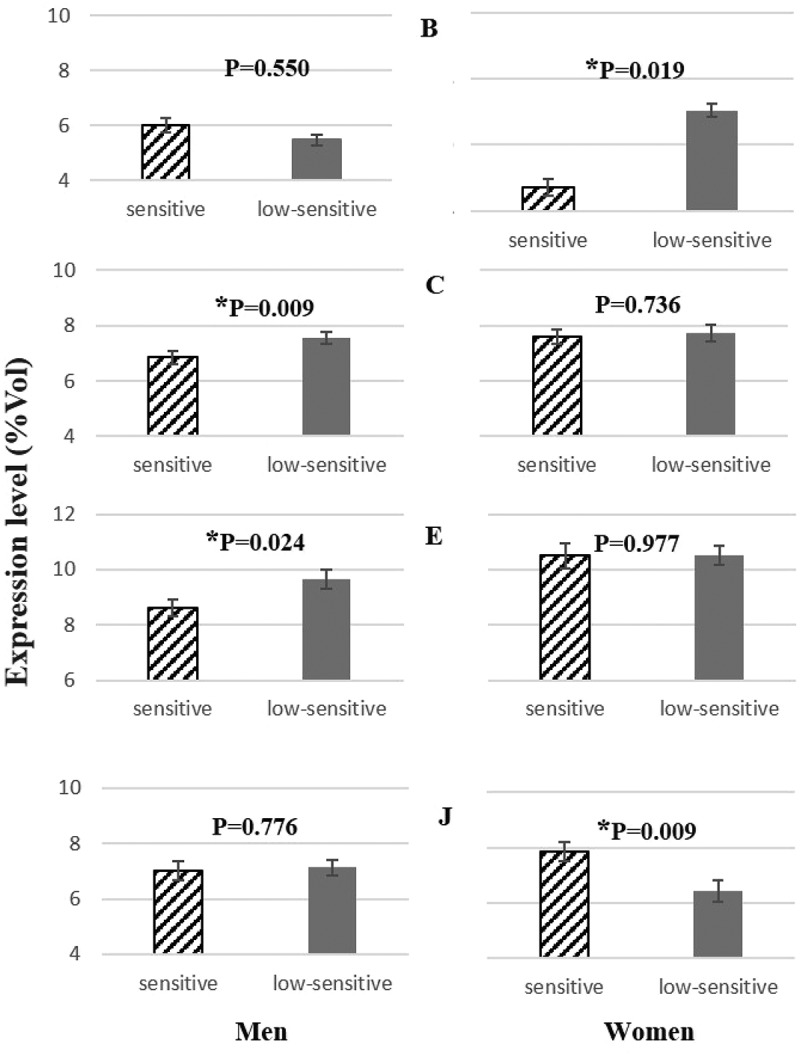
Protein bands differing between the two sweet sensitivity groups. * Differences are statistically significant for P < 0.05.

**Figure 2.**
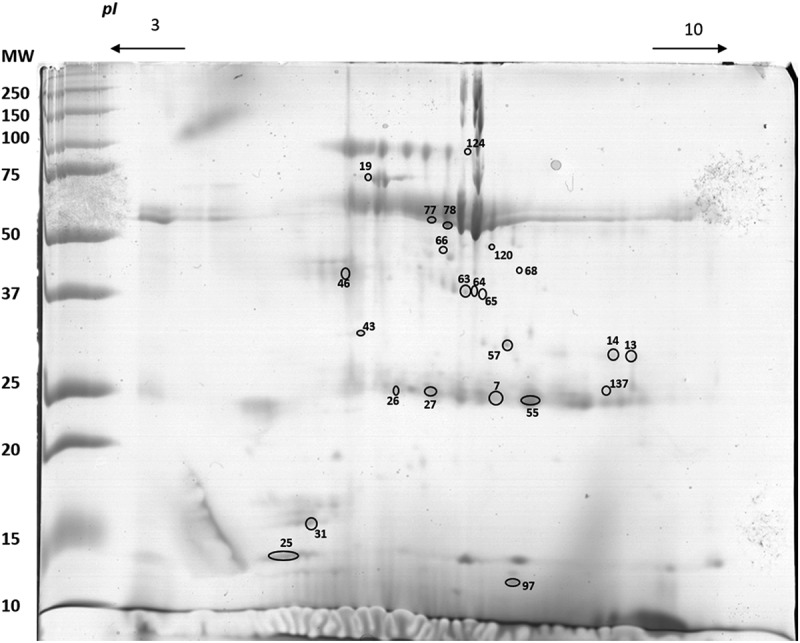
Representative 2-DE profile of saliva analysed. Circles represent the spots differentially expressed between sensitive and low-sensitive groups. MW – molecular weight (kDa); pI – isoelectric point.

SDS-PAGE electrophoresis allows us to demonstrate differences in salivary protein profiles between the two sweet taste sensitivity groups, although these differences were sex-dependent: 1) low-sensitive males had higher levels of expression of protein bands identified as containing polymeric immunoglobulin receptor (Band C) and salivary α-amylase (Band E) (Figure 1); 2) in females a protein band with apparent molecular weight of 120 kDa (Band B), which was not identified by mass spectrometry, was present in higher levels in low-sensitive, compared to sensitive, individuals; conversely, the band containing cystatin-SN (Band J) had lower expression levels in low-sensitive females (Figure 1). Details of the bands identified by mass spectrometry are presented in Table 3.

**Table 3.** MS identification of the salivary proteins differentially expressed among taste sensitivity groups.

Band	Protein	Uniprot entry reference	Estimated/theoretical MW (kDa)	Id score	Sequence coverage (%)	No. peptides matched
B	*Not identified*	–	120.0/–	–	–	–
C	Polymeric immunoglobulin receptor	P01833	84.9/84.4	107	21	13
E	Alpha-amylase 1	P04745	66.5/57.8	135	31	15
F			57.1/57.8	100	21	9
J	Cystatin-SN	P01037	14.4/16.6	99	60	7

Results of 2-DE gel analysis are presented in Table 4, with details of the proteins differently expressed between sweet taste sensitivity groups that were identified by mass spectrometry. A total of 23 protein spots differed between groups (Figure 2), although some of them presented differences in only one of the sexes (eight in males and 10 in females). The common outcomes for males and females are the lower levels of expression of salivary α-amylase spots (66, 77, 78 and 120) in the sweet-sensitive individuals and the higher levels of cystatins (spots 25 and 97) in this group. Although the α-amylase spots that differed between sweet sensitivity groups were not the same in males and females, in each of the sexes a positive correlation was observed between the expression levels of these spots and the detection thresholds of sucrose (spot 66: R = 0.656, *p* = 0.001 in males; spot 77: R = 0.575, *p* = 0.048, in females). Among the proteins identified as belonging to the cystatin family, cystatin S (spot 25) was negatively correlated with sucrose detection thresholds in males (R = −0.567; *p* = 0.009).

**Table 4.** Details of mass spectrometry results for protein spots associated with sweet taste sensitivity. Comparisons between sweet taste sensitivity groups are presented.

*Spot*	Protein	Uniprot entry reference	Estimated/theoretical MW (kDa)	Estimated/theoretical pI	Mascot ID *Score*	Seq. C (%)	No. peptide matched	% Volume (mean ± standard error)		
								Sensitive	Low-sensitive	P
***Men + Women***										
25	Cystatin-S	P01036	13.4/16.5	4.9/4.95	127	67	8	9.05 ± 0.92	4.55 ± 0.84	0.002
63	Carbonic anhydrase 6	P23280	38.6/35.5	5.9/6.51	71	19	5	0.22 ± 0.05	0.39 ± 0.04	0.013
64			37.8/35.5	6/6.51	55	19	5	0.14 ± 0.02	0.21 ± 0.02	0.031
65			37.1/35.5	6.5/6.51	94	30	8	0.07 ± 0.007	0.09 ± 0.009	0.032
***Men***										
7	Ig kappa chain C region	P01834	26.4/11.8	6/5.58	44	48	3	0.27 ± 0.06	0.46 ± 0.06	0.022
13	Cysteine-rich secretory protein 3	P54108	31.0/28.5	7.0/8.09	51	17	4	0.14 ± 0.02	0.24 ± 0.03	0.005
19	Serum albumin	Q86YG0	73.7/71.3	5.6/5.92	132	28	15	0.95 ± 0.16	0.49 ± 0.09	0.015
25	Cystatin-S	P01036	13.4/16.5	4.9/4.95	127	67	8	11.09 ± 1.30	5.27 ± 0.25	0.010
46	Actin cytoplasmic 1	Q96HG5	55.9/42.1	5.4/5.29	121	42	11	0.16 ± 0.03	0.29 ± 0.05	0.035
63	Carbonic anhydrase 6	P23280	38.6/35.5	5.9/6.51	71	19	5	0.22 ± 0.03	0.41 ± 0.07	0.028
64			37.8/35.5	6/6.51	55	19	5	0.15 ± 0.02	0.24 ± 0.03	0.042
66	Alpha-amylase 1	P04745	43.6/58.4	5.6/6.47	146	25	13	0.04 ± 0.01	0.08 ± 0.02	0.003
***Women***										
27	Ig kappa chain C region	P01834	25.9/11.8	5.6/5.58	68	50	4	0.41 ± 0.05	0.27 ± 0.02	0.018
55			25.9/11.8	6.5/5.58	80	50	4	2.70 ± 0.35	1.78 ± 0.21	0.023
31	Polymeric immunoglobulin receptor	P12273	16.4/16.8	5.2/8.26	154	55	9	0.21 ± 0.06	0.39 ± 0.06	0.037
65	Carbonic anhydrase 6	P23280	37.1/35.5	6.5/6.51	94	30	8	0.07 ± 0.01	0.11 ± 0.01	0.008
77	Alpha-amylase 1	P04745	49.7/58.4	5.7/6.47	147	38	17	0.24 ± 0.03	0.36 ± 0.03	0.013
78			59.4/58.4	5.7/6.47	138	35	16	1.62 ± 0.22	2.38 ± 0.26	0.036
120			49.6/58.4	6/6.47	177	32	13	0.07 ± 0.01	0.11 ± 0.02	0.051
97	Cystatin-B	P04080	12.11/11.2	5.6/6.96	76	55	5	0.65 ± 0.16	0.18 ± 0.02	0.018

MW – molecular weight (kDa); *pI –* isoelectric point; (–) Missing values due to unsuccessful identification.

Carbonic anhydrase VI was identified in three of the spots (63, 64 and 65) that were at higher levels in low-sensitive individuals. Two of them differed in males (spots 63 and 64) and one in females (spot 65). Ig K chain C region also differed between sweet taste sensitivity groups in both males and females. However, the spot that expressed differently in males (spot 7) was increased in low-sensitive individuals, whereas, in contrast, the spots expressed differently in females (spots 27 and 55) were increased in the sweet-sensitive group.

Spots 14, 26 and 57, which were observed at higher levels in low-sensitive individuals (considering males + females) failed identification by mass spectrometry. The same happened for spots 68 and 137, also increased in low-sensitive individuals, but only in males, while spots 43 and 124 decreased and increased, respectively, only in females.

To evaluate whether BMI has an effect on the relationship between salivary proteins and sweet taste sensitivity, a two-way analysis of variance was used. No effect due to BMI was observed at the level of any protein spot. Although the number of individuals from the overweight group analysed by 2DE was relatively low (*n* = 9), it appears that BMI does not have a major influence on the relationship between salivary 2-DE protein profile and sweet taste sensitivity.

#### Salivary α-amylase enzymatic activity

**Figure 3.**
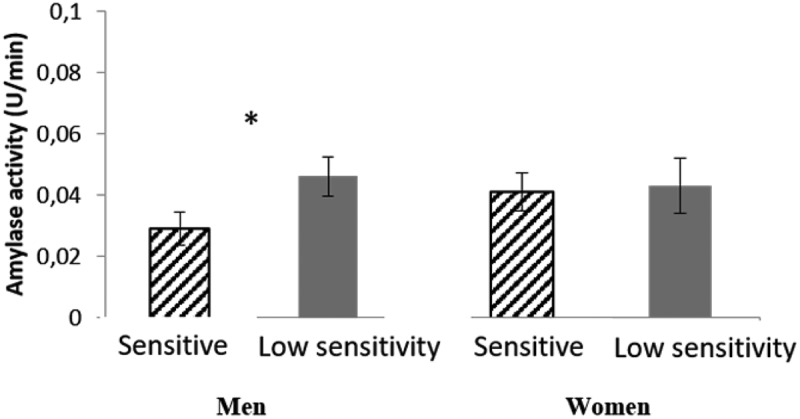
Enzymatic activity of salivary α-amylase (U/min) in men (N = 46) and women (N = 41) with different sensitivity levels to sweet taste (mean ± SEM). *Statistically significant differences: P < 0.05.

The specific enzymatic activity of salivary α-amylase (U/mL) did not present significant differences between sweet taste sensitivity groups, when considering both males and females together. However, making the analysis separately for each sex and taking into account each individual salivary flow rate [converting into enzymatic activity per unit of time (U/min)], it was observed that males who are low-sensitive to sweet taste had higher enzymatic activity per minute. This was not observed in females (Figure 3).

#### Evaluation of salivary cystatin S-SA-SN and CA VI by Western blot

Western blotting for cystatin S-SA-SN and CA VI was performed since, apart from amylase, these were proteins identified in gel bands and/or spots differing between sensitive and low-sensitive individuals.

**Figure 4.**
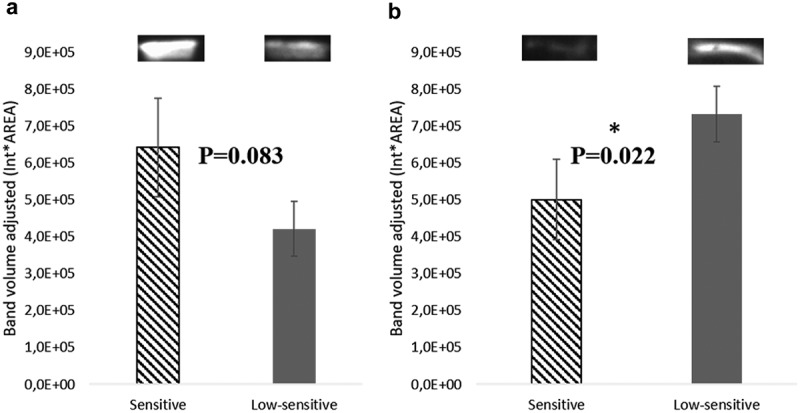
Representative Western blot analysis of cystatins S-SA-SN (a) and CA VI (b) in mixed saliva samples of individuals with different sensitivity levels to sweet taste (mean ± SEM). *Statistically significant differences: P < 0.05.

With regard to cystatin S-SA-SN, differences were observed only in males, for whom this protein showed a tendency to be present in higher salivary levels in individuals sensitive to sweet taste (sensitive: 642,029 ± 133,605, low-sensitive: 421,278 ± 74,119; *p* = 0.083) (Figure 4(a)). Differences in salivary CA VI were also observed only in malese, but in this case it was the low-sensitive ones that presented the higher levels of this protein (sensitive: 501,162 ± 109,733; low-sensitive: 732,072 ± 75,175; *p* = 0.022) (Figure 4(b)).

## Discussion

The objective of this study was to analyse the relationship between saliva composition and sweet taste sensitivity and to assess whether this is influenced by BMI. To date, most studies have addressed the involvement of salivary proteins in the perception of astringency or bitter taste. To our knowledge, this is the first study where the relationship between saliva proteome and taste sensitivity to sweetness has been evaluated.

Taste perception has been referred to as being potentially influenced by factors such as sex [20] or BMI [21]. In the present study the proportions of individuals belonging to sensitive or low-sensitive groups have been observed to be similar in normal weight and overweight groups. However, comparing sexes, a tendency was observed for higher sweet taste sensitivity in females compared to males. An increased taste acuity in females compared to males had been already reported by other authors [20], supporting our results.

A relationship between salivary glucose levels and sensitivity to sweet taste was observed in the subjects evaluated in the present study. This relationship was evident only in females, and saliva from the ones who were low-sensitive to sweetness presented a higher concentration of glucose compared to females sensitive to this taste. One hypothesis to explain these results may be a higher continuous stimulation of taste receptors in the females who have higher salivary glucose concentrations. This may lead to a desensitization of these receptors, resulting in the need for higher concentrations of sweet stimuli to be perceived. Desensitization of taste receptors due to constant stimulation has previously been reported [22].

Through both SDS-PAGE and 2-DE electrophoretic protein profiles it was possible to demonstrate the existence of a relationship between salivary proteome and sensitivity to sweet taste: several salivary proteins were present in different levels according to sweet taste sensitivity and some of these proteins differed in only one of the sexes. Salivary α-amylase was inversely related to sweet taste perception, with higher levels of this protein in the individuals presenting higher sucrose threshold detection, i.e. lower sweet taste sensitivity. Although the protein bands/spots observed to be related to sensitivity to sweet taste were not the same in males and females, and although it was only in males that the relationship between this taste sensitivity and enzyme activity reached statistical significance, the common feature for both sexes was that individuals with low sensitivity to sweet taste had higher mean salivary levels of this protein. The salivary α-amylase protein has the main function of cleaving glycosidic bonds of complex carbohydrates, thus initiating digestion of the starch in the mouth [23]. This results in an increase in the levels of sweet molecules, such as maltose and later glucose, in the oral cavity [24]. Although there were no statistically significant differences in specific enzymatic activity (U/L), a significantly higher enzyme activity per minute (U/min) was observed in males less sensitive to sweet taste. The enzymatic activity per minute takes into account the rate of salivary flow, and can better reflect what happens in the mouth per unit of time. As noted above, for salivary glucose results, higher levels of sweet molecules in saliva may mean a continuous stimulation of the taste receptors, requiring higher concentration of stimuli for sweet perception. The idea that a low sweet taste perception may be associated with higher levels of salivary amylase is, indirectly, supported by other observations: obese individuals, either rats [25] or humans [17], have increased levels of this salivary protein; at the same time, they appear to have higher preference levels for sweet foods, with the latter suggested as being associated with lower sensitivity to this taste [26].

CA-VI and cystatin salivary proteins were other proteins differentially expressed between sweet sensitivity groups. In general, CA-VI had higher expression levels in the saliva of low-sensitive individuals, whereas cystatins were increased in the individuals sensitive to this taste. Although its complete function remains to be elucidated, salivary CA-VI protein has been reported as a trophic factor in the growth and development of taste buds [27] and deficiencies in this protein have been associated with diminished gustatory perception [28]. In this context, higher levels of CA-VI could be expected in sensitive individuals rather than in low-sensitive subjects. However, the relationship of this protein with taste sensitivity is not a matter of consensus, with some authors reporting no association between its levels and taste perception [29]. Moreover, the association of this protein with taste has been mainly studied in terms of bitter taste perception [6,30]. Although the transduction of sweet and bitter tastes shares common mechanisms [31], it is possible that they are affected differently by saliva. As has been previously mentioned, until now most of the authors have studied the relationship between saliva composition and taste in terms of bitter taste. Further studies are needed to understand the different influence of salivary proteins in sweetness.

Cystatins from types S and B were observed to be present at higher levels in the saliva of individuals sensitive to sweet taste, in the present study. These proteins, which are inhibitors of cysteine proteases and participate in the control of proteolysis within the oral cavity [32] have been previously related with bitter taste perception [7,10]. In the latter case, the individuals with the lowest sensitivity to bitter taste were those with a greater abundance of cystatins in saliva. The proposed explanation for this is that high levels of expression of cysteine protease inhibitors in saliva could result in low levels of proteolysis, which might affect the mucosal lining of the oral cavity, reducing the accessibility of the taste molecules to the respective receptors [7]. Since sweet taste perception also needs molecules to reach protein receptors at the membrane of taste cells, we might expect that individuals with higher levels of salivary cystatins would be low-sensitive to sweet taste. However, we observed the opposite in the present study, particularly in the case of males. It is also relevant to point out that the forms of cystatins that were related to sweet taste sensitivity in males and females were not the same: whereas in males it was type-S cystatins that differed between groups, in females the variations were mainly at the level of cystatin B. The presence of these forms in saliva appears to derive from different origins and may have different actions: cystatins of S-type are mainly secreted by submandibular salivary glands [33], whereas cystatin B derives mainly from plasma [34]. As such, and similarly to what was stated for CA VI, further studies that may elucidate the mechanisms through which these proteins can influence sweet taste perception are necessary. Interestingly, the relationships of each of these proteins with sweet taste sensitivity (observed in the present study) or with bitter taste sensitivity [e.g. [7]] appear to be opposite.

The spots identified as cysteine rich secretory protein 3, region C of the K chain of immunoglobulins and prolactin-induced protein (PIP) are spots that, although being related to sweet taste in both sexes, present a contrasting relationship in males and females. The reason why this happens is not known. With regard to the immunoglobulin K chain (immunoglobulin light chain), higher levels of expression were observed in individuals highly sensitive to bitter taste [7]. Both IgG and IgA are typically present in human saliva [35,36]. The light chain, identified in the aforementioned spots, may belong to any of these immunoglobulin isoforms, so the reason why a different relationship with sweet taste perception occurs in males and females is difficult to explain with existing data. On the other hand, the levels of immunoglobulins present in the saliva are affected by the immunological state of the individuals [36]. Although the individuals tested in the present study declared to be healthy and showed no obvious signs of disease, we could not guarantee that they were free of any type of infection/inflammation affecting the levels of salivary immunoglobulins.

Most of the observed differences in saliva composition, between individuals with different sensitivities for sweet taste, were maintained when considering BMI. In the present study the percentage of overweight individuals was considerably lower than normal weight, and as such the analysis in this last group was performed in a low number of individuals. Moreover, most of the overweight individuals were not obese, but only pre-obese (25 kg/m^2^ < BMI < 30 kg/m^2^). The possibility of having greater differences in the relationship between saliva and sweet taste in the obese is not to be ignored and needs to be further elucidated.

Apart from salivary amylase, for which the enzymatic activity may have a direct effect in the levels of sweet molecules present in the mouth, as mentioned above, the relationship between salivary proteins and sweet taste sensitivity may be explained by more than the specific function of each individual protein. It may be hypothesized that salivary proteins with charge near neutrality, at the pH of saliva, might precipitate and/or complex glucose, making its access to taste receptors difficult. Although this theory needs to be further confirmed, looking at the results obtained for males, where salivary glucose levels did not correlate with sensitivity to sweet taste, the observation of higher levels of proteins whose pI is close to the pH of saliva, in low-sensitive individuals, supports the idea of possible interaction of these salivary proteins with glucose molecules. By contrast, in females the pI of the protein spots increased in low-sensitive individuals were not particularly close to that of saliva pH and, in this sex, glucose concentrations were related to sweet taste sensitivity.

It is interesting to note the different relationship that sweet taste sensitivity has with saliva composition in males and females, as has been discussed previously. Some of these differences may be due to hormonal factors. In the present work, there was no control in relation to the female menstrual cycle. Sucrose detection thresholds have been observed to differ between the various phases of menstrual cycle [37]. Moreover, saliva flow rate and protein composition, including salivary amylase activity, were observed to be higher in the ovulatory and luteal phases [38]. This potential influence of the hormonal cycle on our results needs to be elucidated in future studies.

## Conclusion

Although in recent years some studies have related the salivary proteome to oral perception, they were focused on astringency or bitter taste. This might be due to the often-accepted idea that the response to PROP and PTC compounds reflects sensitivity to different oral sensations. However, the results presented in this chapter show that the composition of saliva is also related to sweet taste sensitivity. Curiously, some of the proteins involved in sweet taste sensitivity, such as cystatins and CA-VI, had been previously associated with bitter taste, but in the opposite direction, which reinforces the need for an in-depth investigation of the meaning of salivary proteins in each type of basic taste perception. Salivary glucose levels appear to be related to taste sensitivity, but only in females. However, this sex-related effect of salivary glucose levels in sweet taste sensitivity, together with the observation that males low-sensitive to this taste have higher levels of salivary proteins with isoelectric points closer to the pH of saliva, suggests that proteins may interfere in glucose transport to taste receptors, influencing sweet taste perception.

To our knowledge, this is the first study in which the salivary proteome is compared among individuals with different levels of sensitivity to sweet taste. These first results support the hypothesis of a relationship between saliva and perception for sweet taste, which will need further study, in order to understand the mechanisms of this fluid at the level of this taste perception.
